# ZH-ECochG Bode Plot: A Novel Approach to Visualize Electrocochleographic Data in Cochlear Implant Users

**DOI:** 10.3390/jcm13123470

**Published:** 2024-06-14

**Authors:** Marlies Geys, Leanne Sijgers, Ivo Dobrev, Adrian Dalbert, Christof Röösli, Flurin Pfiffner, Alexander Huber

**Affiliations:** Department of Otorhinolaryngology, Head & Neck Surgery, University Hospital Zurich, University of Zurich, 8091 Zurich, Switzerland

**Keywords:** cochlear implant, electrocochleography, auditory evoked responses, neurophysiological monitoring, audiological visual representation

## Abstract

**Background:** Various representations exist in the literature to visualize electrocochleography (ECochG) recordings along the basilar membrane (BM). This lack of generalization complicates comparisons within and between cochlear implant (CI) users, as well as between publications. This study synthesized the visual representations available in the literature via a systematic review and provides a novel approach to visualize ECochG data in CI users. **Methods:** A systematic review was conducted within PubMed and EMBASE to evaluate studies investigating ECochG and CI. Figures that visualized ECochG responses were selected and analyzed. A novel visualization of individual ECochG data, the ZH-ECochG Bode plot (ZH = Zurich), was devised, and the recordings from three CI recipients were used to demonstrate and assess the new framework. **Results:** Within the database search, 74 articles with a total of 115 figures met the inclusion criteria. Analysis revealed various types of representations using different axes; their advantages were incorporated into the novel visualization framework. The ZH-ECochG Bode plot visualizes the amplitude and phase of the ECochG recordings along the different tonotopic regions and angular insertion depths of the recording sites. The graph includes the pre- and postoperative audiograms to enable a comparison of ECochG responses with the audiometric profile, and allows different measurements to be shown in the same graph. **Conclusions:** The ZH-ECochG Bode plot provides a generalized visual representation of ECochG data, using well-defined axes. This will facilitate the investigation of the complex ECochG potentials generated along the BM and allows for better comparisons of ECochG recordings within and among CI users and publications. The scripts used to construct the ZH-ECochG Bode plot are provided by the authors.

## 1. Introduction

The last two decades have shown a proliferation of studies investigating the use of electrocochleography (ECochG) in cochlear implant (CI) recipients. ECochG is a method of measuring electrophysiological responses to acoustic stimuli, capturing the early potentials generated by the cochlea and auditory nerve fibers. The ECochG response consists of four components, known as the cochlear microphonic (CM), auditory nerve neurophonic (ANN), compound action potential (CAP) and summating potential (SP) responses. The CM is mainly a hair cell potential, whereas ANN and CAP are neural potentials; the SP has contributions from both hair cells and neural sources [1,2,3,4,5,6]. The responses can be measured at both extracochlear and intracochlear sites, whereby the latter is now achievable through direct recordings with the CI’s electrode array. ECochG recordings in cochlear implants have demonstrated potential relevance in monitoring cochlear function during and after insertion of the CI electrode [7,8,9,10,11,12,13,14,15,16,17,18,19], detecting intracochlear trauma [20,21,22,23], optimizing the electrode’s placement [24,25], estimating hearing thresholds [26,27,28,29], investigating different etiologies of hearing loss [30,31,32,33], and predicting postoperative preservation of hearing [12,34,35,36,37,38,39,40,41,42] and CI speech perception outcomes [43,44,45,46,47,48,49,50].

When measuring ECochG responses along the basilar membrane (BM), the tonotopic recording location is of interest to better understand these cochlear responses. Because of the different types of commercially available electrode arrays (e.g., straight or precurved electrodes), electrode array lengths, surgical approaches (e.g., round window (RW) or cochleostomy), and surgical insertion depths, the angular insertion depth (AID) will vary among CI recipients. Additionally, the final cochlear coverage of the CI can differ among individuals, even with the same types of electrode array, due to anatomic variations in the length of the cochlear duct and the cochlear diameter (for a review, see [51]). These all lead to a unique AID within the cochlea of the CI recipient, which needs to be considered when investigating ECochG responses at various recording locations along the BM.

However, different visual representations of ECochG responses exist in the literature, making it difficult to compare ECochG recordings among various publications. For example, intracochlear recordings using the CI’s electrode contacts are mostly visualized showing the amplitude of the response at the different recording electrodes. The locations of these contacts vary, for example, due to different numbers of contacts in the CI electrode arrays. Additionally, the F0 amplitude of the ongoing CM response is frequently visualized without any additional information such as the preoperative audiometric profiles, CM latency shifts, or neural contributions, although this information is important for the interpretation of ECochG recordings [12,18,29].

This study explored the different visual representations available in the literature in a systematic review and proposes a novel approach to visualize recorded ECochG data in CI users. The new visual representation aims to facilitate comparisons of ECochG recordings among publications and within individuals by providing all the necessary information in a standardized format in one graph.

## 2. Materials and Methods

### 2.1. Systematic Review

To provide an overview of the existing visualization methods of ECochG recordings in CI recipients, a systematic review was conducted. The Preferred Reporting Items for Systematic Reviews and Meta-Analyses (PRISMA) guidelines [52] were followed, to the best of the authors’ ability, for the relevant sections and topics. Two databases (PubMed and EMBASE) were queried up to 10 April 2024 for the following terms: “Electrocochleograph*” OR “ECochG” OR “ECoch” OR “ECoG” OR ”cochlear microphonic*” OR “auditory nerve neurophonic*” AND “cochlear implant*”. An asterisk (*) was used as truncation to allow multiple endings. The terms needed to be present in the title or abstract. The inclusion criteria were articles written in English, available as a full text, and including at minimum one figure that displayed ECochG recordings at various locations along the BM performed in a cochlear implant recipient or a related animal study. The focus of the systematic review was the visualization of the ongoing portion of the response waveform at intracochlear recording sites. Extracochlear measurements were also included if the ECochG responses were measured over multiple steps of insertion. One author (M.G.) screened the figures of each article and excluded those with no figures or those that were irrelevant to the purpose of this review. No automation tools were used. The figures were analyzed according to the following parameters: (1) time of recording (intra- or postoperative), (2) location of the recording (extra- or intracochlear), (3) x-axis used, (4) y-axis used, and (5) the ECochG component (e.g., CM). Furthermore, the representation of pre- and postoperative audiograms, the use of more than two axes (e.g., amplitude and phase), the visualization of multiple curves on a single graph (e.g., multiple frequencies), and the use of subplots were documented. Multiple figures from the same publication were included if the parameters described above varied. The review’s protocol was not registered.

### 2.2. Construction of the ZH-ECochG Bode Plot 

A novel visualization of individual ECochG data (the ZH-ECochG Bode plot) was developed after assessing the currently used visualization methods using the abovementioned systematic review. The ZH-ECochG Bode plot, where ZH stands for the region of Zurich, where the plot was created, should convey the following: (1) the ECochG response’s amplitude and phase with respect to the recording electrodes’ tonotopic position and insertion angle, (2) pre- and postoperative audiometric profiles for a comparison of the ECochG responses along the BM, and (3) a comparison of multiple ECochG measurements within a CI recipient. The ZH-ECochG Bode plot was demonstrated using ECochG recordings in three CI recipients who received different CI systems. Participant P1 received a CI with a MidScala electrode array with 16 intracochlear electrode contacts (Advanced Bionics LLC, Valencia, CA, USA); the other two participants received a CI622 (participant P2) and CI612 (participant P3) electrode array (both from Cochlear Limited, Sydney, Australia). Recordings were obtained using the standard CI software (Cochlear^TM^ Research Platform (v1.2) and AIM^TM^ System (OM Suite v1.1)) and hardware provided by the CI manufacturer. For each CI recipient, measurements were performed while stimulating them with a 500 Hz tone burst (1) during continuous insertion of the electrode array, (2) immediately after complete insertion of the electrode array, and (3) around 6 weeks following CI surgery. For the measurements performed during insertion of the electrode array (Measurement 1), hereafter referred to as “insertion monitoring”, the CI’s most apical electrode was used to record the ECochG responses. The time points of the electrode’s contacts entering the cochlea were monitored during surgery. For the measurements performed after insertion of the CI, hereafter referred to as intra- or postoperative electrode sweeps (Measurements 2 and 3), each individual intracochlear electrode’s contact (for CIs from Advanced Bionics LLC) or every second electrode’s contact (for CIs from Cochlear Limited) was used to obtain ECochG recordings. Pure-tone audiometry was conducted within 6 months prior to surgery and within 3 months after cochlear implantation, in accordance with ISO 8253-1:2010 [53]. The air conduction threshold values were determined at 0.25, 0.5, 1, 2, 3, 4, 6, and 8 kHz. The maximum output of the audiometer plus 5 dB was used as a threshold value if no response was present at the maximum output of the audiometer. Postoperative hearing preservation (HP) was calculated using the HEARRING Group’s classification system, with 100% indicating complete HP and 0% indicating a total loss of hearing [54]. The study was approved by the local ethics committee (KEK-ZH-2020-00639) and conducted in accordance with ICH-GCP (Good Clinical Practice) guidelines and the Declaration of Helsinki.

**Figure 1:**
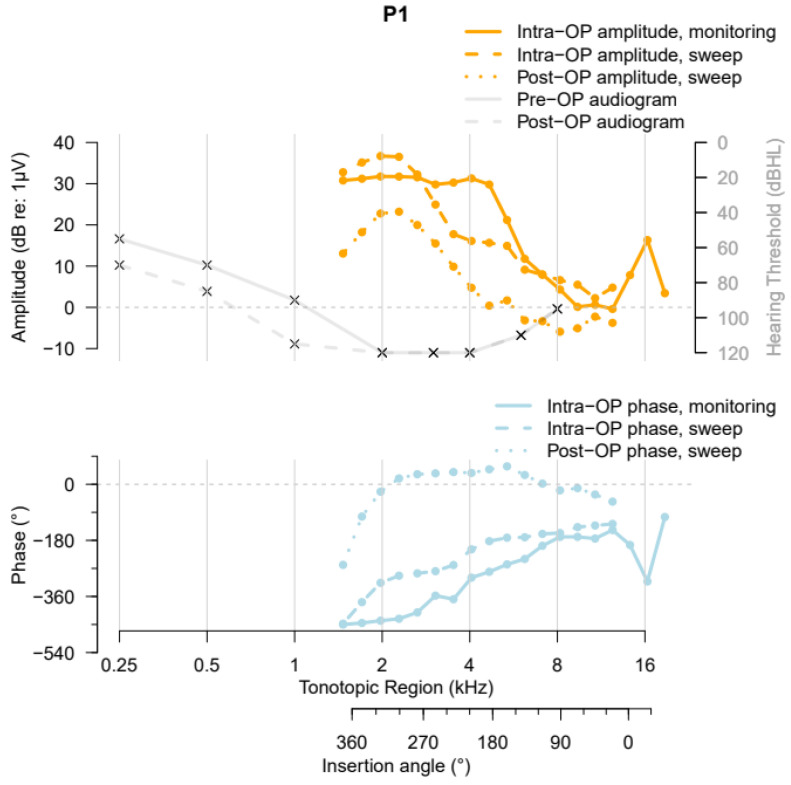
Exemplary ZH-ECochG Bode plot, using data from study participant P1, who received a MidScala electrode array. The upper part shows the pre- and postoperative audiograms and the amplitudes of the ECochG responses under stimulation with a 500 Hz tone burst recorded at different time points, plotted against the cochlea’s tonotopic region and the CI’s insertion angle. The lower part shows the corresponding ECochG phases, plotted against the same *x*-axis as the upper part of the figure.

Figure 1 shows the ZH-ECochG Bode plot using data from study participant P1. The upper graph includes the pre- and postoperative audiograms, with the different hearing thresholds (right-hand *y*-axis, decibels hearing level (dB HL)) at the tested frequencies and the F0 amplitudes of the different responses (left-hand *y*-axis, dB relative to 1 µV) at the different insertion angles in degrees (°). Different measurements performed in the same participant were visualized on the same axes to enable the comparison. A horizontal line was drawn at 0 dB re:1 µV, as lower amplitudes are commonly interpreted as no CM response. The lower part of the figure demonstrates the phase changes occurring (left-hand *y*-axis, ° relative to the acoustic stimulus) at different insertion angles (°). Similar to the upper part of the figure, different measurements in the same participant were visualized on the same axes. The R (v4.3.1) script used to construct the graph, together with the example of data from participant P1, is provided at https://github.com/OtoBM.

While such graphical representation facilitates an objective comparison of ECochG data from various CI electrode arrays, the derivation of the recording electrode’s tonotopic region and insertion angle, which are used to construct the ZH-ECochG Bode plot, is non-trivial. We propose a method for determining these two *x*-axes based on the electrode’s position and the length of the cochlear duct assessed using postoperative CT (computer tomography) imaging, and we created a MATLAB (R2022b) script to do so. Since postoperative imaging is not available in all clinics, and the analysis of postoperative CT scans is time-consuming, we also provide standardized *x*-axes for several commonly used CI electrode arrays from MED-EL (Innsbruck, Austria), Advanced Bionics LLC (Valencia, CA, USA), and Cochlear Limited (Sydney, Australia).

**Figure 2:**
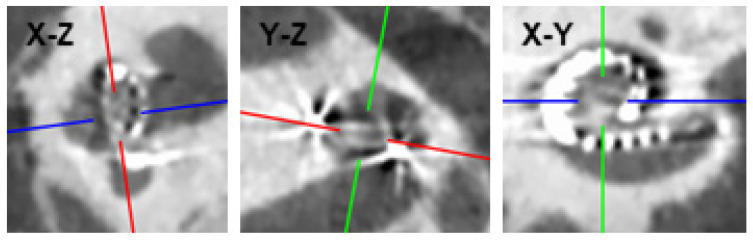
Example of a CT scan of a cochlea with an inserted MidScala electrode array (P1). The red lines in the *x-z* and *y-z* planes are aligned with the cochlea’s basal turn so that the *x-y* plane is aligned with the cochlea. One or more slices in the *x-y* plane, showing the CI electrodes’ contacts, can then be exported for analysis.

For defining the *x*-axes using postoperative imaging, the three-dimensional postoperative computed tomography (CT) scans were overlaid on a two-dimensional plane. This was achieved by performing a rigid transformation of the scan using the hospital’s clinical software (Synedra AIM (v23), Synedra Information Technologies GmbH, Innsbruck, Austria) to align the *x-y* plane with the cochlea’s basal turn (Figure 2, left and middle). Next, one or more slices in this plane that showed the CI’s electrodes were selected and exported as figures (Figure 2, right). In the case when multiple slices were needed to visualize all electrodes, the exported slices were overlaid to obtain one image showing all the intracochlear electrodes’ contacts. A Python (v3.9) script to overlay the different images is provided at https://github.com/OtoBM, but other software can be used for this purpose as well, including image editing software.

**Figure 3:**
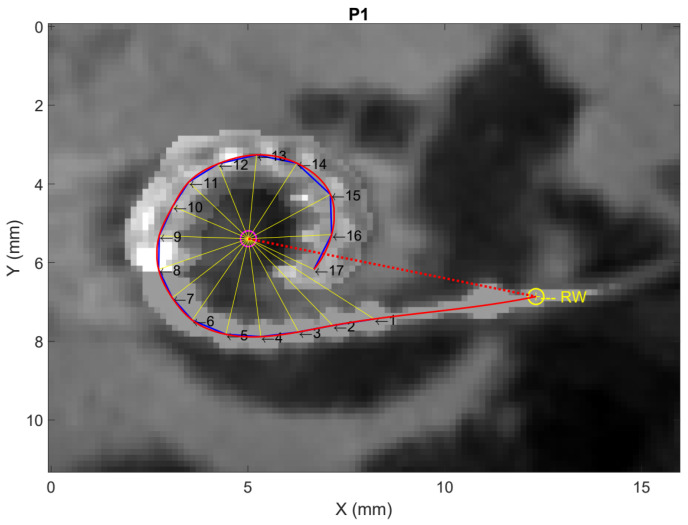
Visualization of the method used to extract the individualized insertion angles and tonotopic regions for each recording location of the ECochG electrode sweep of participant P1. The pink and yellow circles, respectively, show the cochlear center and round window (RW), while the arrows, denoted by numbers 1 to 17, show the various points along the electrode array selected by the user. The solid blue lines show the linear connections between the selected points, while the solid red line shows the trajectory of the electrode array estimated using spline interpolation. The dashed red line connects the RW and cochlear center, and the thin yellow lines connect the selected points along the array with the cochlear center.

The resulting overlaid image can then be used to extract the individualized insertion angles and tonotopic regions for each recording location of the intra- and postoperative electrode sweep, using a MATLAB (R2022b) script provided at https://github.com/OtoBM. The script allows the user to insert the length of the CI recipient’s cochlear duct, in the case that this has been determined clinically using separate image analysis software, or to use the default value of 36.2 mm [55]. The user is then asked to select the cochlear center and the round window in the overlaid image, followed by manual selection of several points along the electrode array, including the first and last electrodes’ contact (Figure 3). The trajectory of the electrode array is estimated by interpolation of one-dimensional data with MATLAB R2022b’s interp1 function using the ‘spline’ method, based on the selected round window and the points along the electrode array. The interpolation returns 10^6^ equally spaced points, and the insertion depth of the most apical electrode (in mm) is derived by adding consecutive interpolated points up to that electrode’s location. The insertion depths of all other electrodes are derived by linear interpolation using the apical electrode’s insertion depth, the number of electrodes, and length of the electrode array, as specified in [56]. In case this calculation results in a negative insertion depth for the basal electrode, the respective insertion depths of the apical and basal electrode are set to the array length and zero, respectively, and linear interpolation is used to calculate the insertion depths of all other electrodes. The recording locations for the insertion monitoring data are derived from the sweep data’s axes using linear extrapolation up to an insertion depth of 0 mm, as shown in Figure 1.

The tonotopic regions are calculated from the electrodes’ insertion depths and the length of the cochlear duct using the Greenwood function [57]. To calculate the electrodes’ AIDs, initially, the angles between the thin yellow lines and the dashed red line in Figure 3 are derived. This gives the insertion angles of all points along the electrode array that were selected by the user, including the apical and basal electrode. The AIDs of the individual electrodes are then derived by linear interpolation between the apical and basal electrode contacts’ insertion angles. The AIDs for the insertion monitoring data are again derived using linear extrapolation.

In cases where postoperative imaging is not used, the *x*-axes are determined according to a study by Dhanasingh et al. [56]. The electrodes’ insertion depths, in mm, are based on the manufacturer’s specifications, and the corresponding tonotopic regions are derived using the Greenwood function. The electrodes’ AIDs are based on the average measured insertion angles.

## 3. Results

### 3.1. Systematic Review

**Figure 4:**
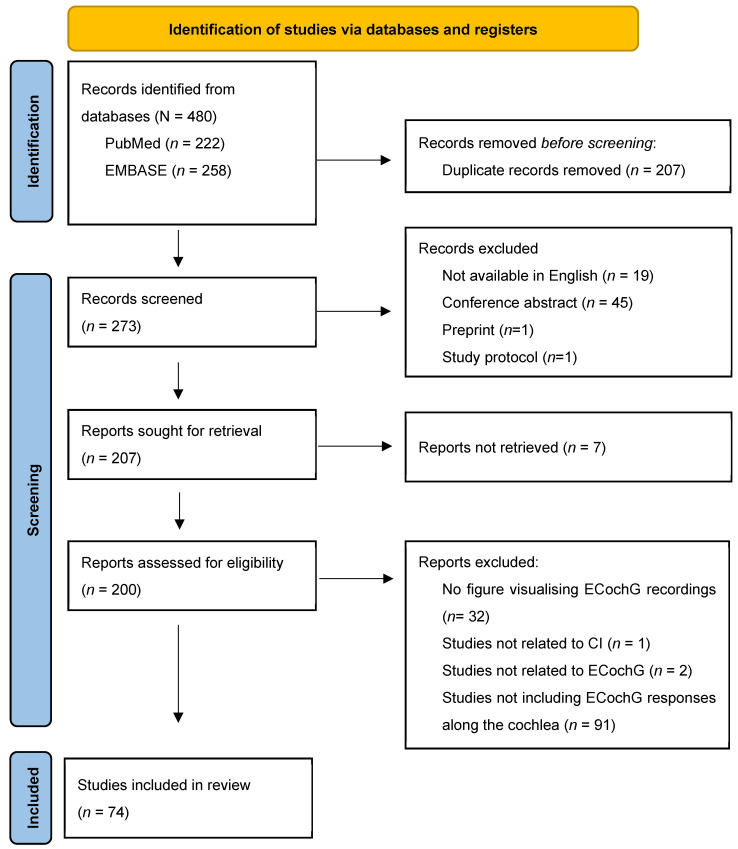
PRISMA 2020 flow diagram. PRISMA, Preferred Reporting Items for Systematic Reviews and Meta-analyses.

In total, 480 records were identified from the different databases (Figure 4). After removing 207 duplicates, another 65 records were eliminated during screening for the following reasons: not available in English (*n* = 19), conference abstract (*n* = 45), preprint (*n* = 1), and study protocol (*n* = 1). Seven reports could not be retrieved, and the remaining 200 reports were assessed for eligibility. Another 125 reports were excluded for various reasons: 32 reports did not include any figure visualizing ECochG recordings, one study was not related to CIs, another two reports used the abbreviation ECoG for “electrocorticography” and were thus not related to ECochG, and finally, the majority of the excluded reports (*n* = 91) did not visualize the amplitude of the ECochG response along different locations in the cochlea. Instead, they showed the growth functions of the amplitude of ECochG, ECochG responses recorded from a single location before and after insertion, or ECochG thresholds for different stimuli, amongst others. The remaining 74 studies [9,10,11,12,13,15,17,18,19,20,21,24,25,34,35,37,39,40,41,42,48,58,59,60,61,62,63,64,65,66,67,68,69,70,71,72,73,74,75,76,77,78,79,80,81,82,83,84,85,86,87,88,89,90,91,92,93,94,95,96,97,98,99,100,101,102,103,104,105,106,107,108,109] were included for review and are described in detail in Table S1 in the Supplementary Materials (https://github.com/OtoBM).

The included articles contained 60 (81.1%) human studies, 11 (14.9%) animal studies, 1 study including both human participants (adults) and animals (guinea pigs), 1 study including only simulated data, and 1 study including both human (adults) and simulated data. Within the human studies, 49 were focused on adults, 2 were focused on pediatric patients, and 9 included both. In the animal studies, four were conducted on gerbils, six on guinea pigs, and one on sheep.

Of the 74 studies included in the review, in total, 115 figures were of interest. Table 1 provides a summary of the 115 figures regarding the different parameters used for the visualization of the ECochG data. The complete set of publications with the corresponding figures and their visualized parameters can be found in Table S1 in the Supplementary Materials (https://github.com/OtoBM).

We found that 85.2% (98/115) of the visual representations plotted ECochG data recorded intraoperatively, 13.0% (15/115) used postoperative data, and 1.7% (2/115) visualized both intraoperative and postoperative recordings in one figure. Within the operating theatre, the majority (70%) of figures visualized the ECochG recordings during insertion of the electrode array. Intracochlear ECochG recordings were visualized in 81% (93/115) of the figures, 10.4% (12/115) showed extracochlear ECochG recordings, and 9.6% (11/115) visualized both recordings in one figure. Of the visualizations showing intracochlear recordings, the majority (89/103) used the CI electrode array for recording ECochG responses along the BM.

In the included figures, different ECochG components were visualized. CM or difference curves (DIF) or the amplitude of the fast Fourier transform at the fundamental frequency (FFT F0) were mostly visualized (61.7%). For the *x*-axis, 34 (29.6%) figures used the insertion time, 23 (20.0%) used the recording electrodes, 19 (16.5%) plotted the time domain of the waveform, 13 (11.3%) visualized the insertion depth, 14 (12.2%) used other axes (e.g., the angle of rotation), and 12 (10.4%) used two *x*-axes. On the *y*-axis, the majority (53.9%) visualized the response magnitude of the ECochG signal, whereas the other figures visualized the raw waveforms’ amplitude (20%), phase (6.1%), and other parameters (2.6%). The remaining figures included two or more *y*-axes (17.4%).

Finally, two figures included the audiogram or audiological data of the participant on the ECochG figure, 16 added notes on the figures during insertion of the CI electrode array (e.g., white marker), 7 included a noise floor, 8 included some additional raw signals in the visualization, 4 used a color bar to visualize the amplitude of ECochG, 66 provided subplots to visualize multiple data (e.g., different participants, different stimulus frequencies) in one figure, 75 added multiple curves in one figure (e.g., different ECochG components), and 2 figures provided a 3D visualization.

### 3.2. The ZH-ECochG Bode Plot in Use

The ECochG recordings of three participants were illustrated using the ZH-ECochG Bode plot with customized axes. Table 2 summarizes the demographic, surgical, and audiological data for the three participants.

Participant 1 (P1) had a typical high-frequency preoperative hearing loss, visualized in Figure 1 (top, solid grey curve), with a pure tone average (PTA) of 83.75 dB HL. Postoperative imaging of Participant 1 revealed that the cochlear duct had a length of 36.2 mm and an AID of 374°. During insertion of the precurved HiFocus MidScala electrode array, a “Harris Type A” ECochG amplitude pattern (i.e., an overall increase in the first harmonic’s amplitude from the beginning of insertion until completion [13]) was measured. A large phase shift was visible in the very early stage of insertion in the basal region of the cochlea. After full insertion and securing of the electrode array, the intraoperative electrode sweep recording showed a peak in the apical region. The highest amplitude was recorded close to the 2 kHz tonotopic region, and the recordings showed a gradual phase delay along the BM. The postoperative ECochG measurement showed a pattern similar to the intraoperative sweep recording, with a slightly larger phase change in the apical region. The HP after cochlear implantation was 57.1%.

**Figure 5:**
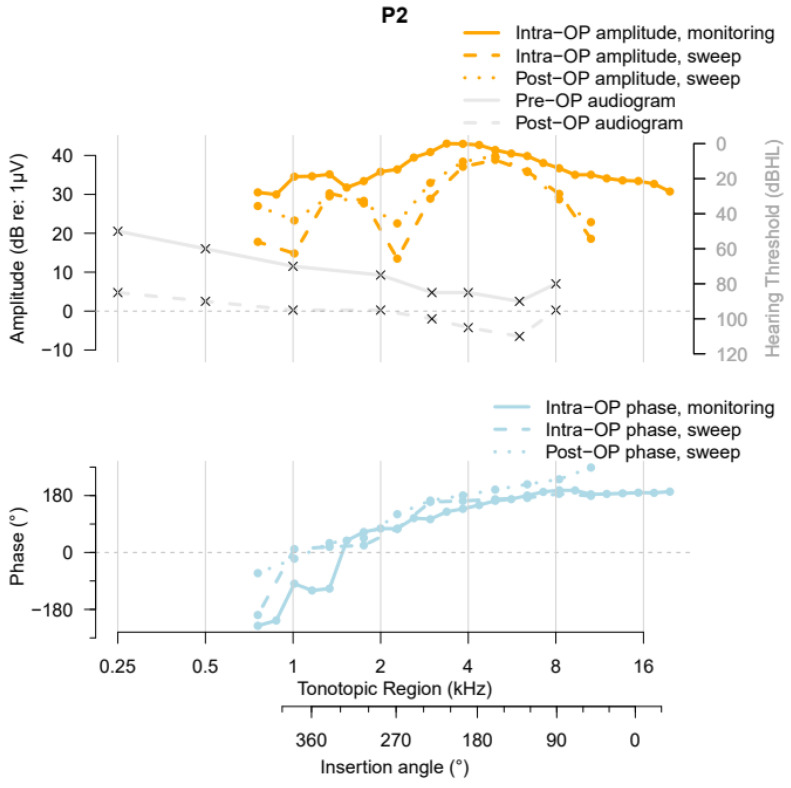
ZH-ECochG Bode plot, using data from study Participant P2, who received a CI622 electrode array. The upper part shows the pre- and postoperative audiograms and the amplitudes of the ECochG responses under stimulation with a 500 Hz tone burst recorded at different time points, plotted against the cochlea’s tonotopic region and the CI’s insertion angle. The lower part shows the corresponding ECochG phases, plotted against the same *x*-axis as the upper part of the figure.

Participant 2 (P2) had preoperative hearing loss (PTA = 63.75 dB HL) due to Ménière’s disease with a more pronounced hearing loss in the high frequencies (solid grey curve, Figure 5). P2 had a cochlear duct with a length of 36.9 mm and an AID of 410°. During insertion of the Slim Straight Electrode CI622, a “Harris Type C” ECochG amplitude pattern (i.e., amplitudes at the start of insertion that were similar to the amplitudes at the completion of the insertion, with a maximum amplitude being reached mid-insertion [13]) was measured. The highest amplitude occurred around the 4 kHz tonotopic region in the cochlea. The phase changes relative to the acoustic stimulus showed an increase in delay along the BM. The intraoperative electrode sweep recording showed a double peak pattern, with the first peak occurring at around an insertion angle of 170° and the second peak at 340°. The double peak pattern and the phase changes stayed stable during the postoperative sweep recording. The HP after cochlear implantation was 37.9%.

**Figure 6:**
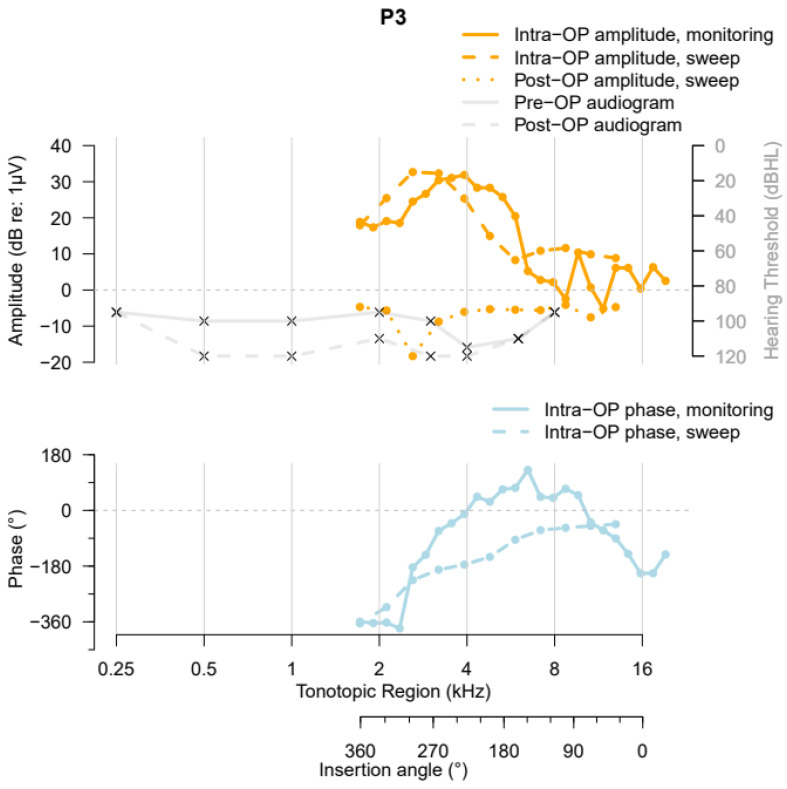
ZH-ECochG Bode plot, using data from study Participant P3 who received a CI612 electrode array. The upper part shows the pre- and postoperative audiograms and the amplitudes of the ECochG responses under stimulation with a 500 Hz tone burst recorded at different time points, plotted against the cochlea’s tonotopic region and the CI’s insertion angle. The lower part shows the corresponding ECochG phases, plotted against the same *x*-axis as the upper part of the figure.

Participant 3 (P3) had little preoperative residual hearing (PTA = 97.5 dB HL) (solid grey curve, Figure 6), a cochlear duct with a length of 37.0 mm, and an AID of 362°. During insertion of the Contour Advance Electrode CI612, a “Harris Type A” ECochG amplitude pattern (i.e., an overall increase in the first harmonic’s amplitude from the beginning of insertion until completion [13]) was measured. The highest amplitude occurred at around the 4 kHz tonotopic region. The phase changes relative to the acoustic stimulus showed an increase in delay after passing the 8 kHz tonotopic region along the BM. The intraoperative electrode sweep recording showed a similar peak pattern, with a slightly more apical peak. Postoperatively, no ECochG recordings above 0 dB (relative to 1 µV) could be recorded. Therefore, the phase data were not visualized in the lower part of the graph. The HP after cochlear implantation was 11.3%, indicating minimal HP.

## 4. Discussion

This study aimed to introduce an innovative visual framework for visualizing ECochG recordings in CI recipients in relation to their precise recording locations along the BM. This novel visualization enhances comparisons across CI users and across various studies, and augments comprehension of ECochG responses within individual CI users.

A review of the literature revealed a diverse range of approaches for visualizing ECochG recordings, which complicated comparisons across different studies. The most common visual representations identified (66.1%) were associated with studies focused on the intraoperative monitoring of electrode insertion, in agreement with the findings in the systematic review by Trecca et al. 2020 [110]. The apical electrode of the CI array was mainly used for recording (43.5%), and the F0 amplitude of the ongoing CM/DIF response was mostly (61.7%) visualized. The surgical time of insertion was mainly used as the *x*-axis (29.6%). Using surgical time on the *x*-axis makes it challenging to analyze the ECochG recordings with respect to the recording location along the basilar membrane. In addition, as the insertion speed varies depending on the type of electrode and the surgeon [111], the scale of the *x*-axis varies between recordings. If a continuous insertion is performed, the corresponding tonotopic regions along the BM can be derived with a certain degree of inaccuracy. This inaccuracy could be overcome by using robotic insertion systems, as they can control the electrode array’s insertion speed [82,96]. Specifying the electrodes’ locations during insertion (e.g., by marking when each electrode’s contact enters the cochlea) can facilitate the post hoc analysis of the recordings, as the approximate location of the recording electrode can be derived from the different markers placed during the measurement. These notes were only included in 16 out of the 115 figures.

Another commonly used visual representation (20.0%) was the ECochG recordings at the different intracochlear CI electrodes during in situ electrode sweeps. As with the commonly used visual representations of ECochG recordings during insertion monitoring described above, the insertion angle and the corresponding tonotopic region for each recording electrode could not be derived from this visualization, given the large variations in insertion depths among different electrode arrays and CI recipients [56,112]. Postoperative imaging or the manufacturer’s specifications of the inserted electrode array are needed to interpret the ECochG results relative to the tonotopic region. Deriving the recording electrodes’ location along the BM was only possible in a minority of the figures when the following *x*-axes were used: insertion depth (11.3%), tonotopic position (2.6%), number of intracochlear electrodes (0.9%), and angle of rotation (0.9%).

Another representation that frequently (20.0%) occurred displayed the raw waveforms with a vertical alignment, corresponding to the different insertion steps or recording sites. This representation allows for the analysis of changes in amplitude, phase delays, and other ECochG characteristics. While this approach complicates the comparison of multiple measurements within or between participants, adding single raw waveforms if desired for clarification of the complex ECochG response can be beneficial

In visualizations showing ECochG characteristics plotted against a measure related to the insertion depth, different ECochG components were illustrated on the ordinate, depending on the research question of the study. The majority of the figures (62.2%) visualized the magnitude of the response or the amplitude of the waveform. Only a few visual representations (6.1%) displayed the phase changes occurring along the BM, and an additional 7.8% showed both the amplitude and phase changes in one figure (i.e., the category with two or more *y*-axes), despite the fact that phase changes can aid the interpretation of fluctuations in the amplitude of ECochG [12,17,113] and increase the predictive power of ECochG responses [67]. Some visualizations (6.1%) included the noise floor to provide information about the reliability of the responses. Moreover, 29.6% of the figures used multiple axes to enhance the displayed information, for instance, integrating the amplitude and phase into a single graph, using a 3D visualization to show the responses elicited by various acoustic stimuli (e.g., waterfall plots), or concurrently showing the insertion depth and surgical duration. In most cases (65.2%), multiple recordings in one or more CI users were shown in one figure, enabling comparisons of different ECochG patterns along the BM.

The ZH-ECochG Bode plot was constructed using the abovementioned advantages and disadvantages found in other visual representations. The novel visual framework uses the insertion angle of each recording site and the corresponding tonotopic region to facilitate a better understanding and comparison of the recorded potentials along the BM. The ZH-ECochG Bode plot shows not only the amplitude but also changes in the phase along the BM to facilitate the interpretation of changes in amplitude and the behavior of the travelling wave along the BM. The direction of the *x*-axis (the tonotopic frequency, given from low to high frequencies) was chosen to be uniform in the well-known Bode plot visualization and the audiogram, implying that the insertion angle, the magnitude of the response during insertion, and the phase changes need to be read from right to left. It also enables the visualization of multiple recordings from a single CI user, allowing monitoring of intracochlear changes over time. Moreover, incorporating the pre- and postoperative audiograms into the plot permits a comparison between the recorded ECochG responses and the audiometric profile. When one compares ECochG recordings with audiograms, it is crucial to exercise caution due to the disparity in the measurements; ECochG amplitudes are elicited by auditory stimuli with a singular intensity and displayed relative to 1 µV, whereas audiograms assess thresholds across various frequencies and are expressed in dB HL, a relative measure.

The goal of the scripts that are made publicly available is to provide a pragmatic application, allowing for future changes if required. The ordinate axis can be changed as desired when different quantification methods of ECochG recordings are used (e.g., ECochG-TR, total response [44]). Additionally, the default length of the cochlear duct of 36.2 mm can be changed, as averages differ between ethnicities and genders [114]. The use of the ZH-ECochG Bode plot can be expanded by adding ECochG responses using different acoustic stimuli (e.g., multifrequency ECochG) or stimulation levels. If desired, important information on the outcome (e.g., the speech reception threshold) can be added in the title of the plot.

It should also be emphasized that the proposed method to derive the *x*-axes on the basis of postoperative imaging provides an approximation of the recording electrode’s tonotopic region and insertion angle, and that the exact values may differ. Image analysis was performed in a two-dimensional plane, while the cochlea itself is three-dimensional (3D). This limitation is partially addressed by the superposition of several planar CT cross-sections that are manually adjusted to be approximately parallel to the individual regions (e.g., first turn, second turn, etc.) of the cochlear duct. Future work should focus on the detailed extraction of the 3D shape of the cochlear duct [115], for example, via automatic labeling of the bony walls of the otic capsule and quantification of its trajectory via 3D skeletonization [116,117,118,119]. This could then be used to map the precise 3D position of the CI electrodes. For calculation of the tonotopic region, the frequencies along the organ of Corti are visualized via the Greenwood function. A tonotopic shift compared with the Greenwood map may occur under high-intensity stimulation [120] and due to the otopathology of the CI users [55].

To estimate the electrodes’ linear insertion depth, from which the tonotopic regions are derived, only the location of the most apical electrode is directly obtained via postoperative imaging; all other estimations of the electrode’s depth are then based on the apical electrode’s location and the manufacturer’s specifications. A correction is implemented in the script if a negative insertion depth for the basal electrode has been estimated, which can occur when the basal electrode contact is close to the round window and an inaccuracy in the calculation of the electrode array length is present due to the manual selection of points along the array in a two-dimensional plane. Future versions should also enable the use of the method for fitting the trajectory of the electrode array for cases with extracochlear electrodes. Since the AIDs are explicitly determined for the apical and basal electrode, and interpolated for all electrodes in between, there is no direct link between the insertion angle and tonotopic frequency axes. For cases where the length of the cochlear duct is accurately determined, the mismatch between the two axes should be minimal.

Another limitation of estimating the electrodes’ location is that the spline interpolation method, used for estimation of the electrode array’s trajectory, will pass through all points selected by the user, regardless of whether there is noise (selection error) in them. Future versions of the script can alleviate this problem by using polynomial fitting or another appropriate function type, as defined in the polar coordinates. Specifically, the cochlear center will be the origin, and the electrode array will be defined as radius–angle pairs of coordinates. This allows for better robustness against user-related selection errors, while adhering to the natural spiral-like shape of the cochlea.

## 5. Conclusions

The ZH-ECochG Bode plot is the first method, to our knowledge, to resolve the lack of generalization in the visualization of ECochG data by using well-defined axes and providing an editable script for visualization. This provides the possibility to better understand the complex ECochG responses recorded at various location along the BM and facilitates comparisons of ECochG recordings within and among CI users and publications.

## Figures and Tables

**Table 1:** Summary of the different parameters (time of recording, recording location, *x*-axis used, *y*-axis used, ECochG component, and extra) visualized in the 115 figures included in the systematic review. The complete set of publications with the corresponding figures and their visualized parameters can be found in Table S1 (Supplementary Materials).

	Parameter	N		Parameter	N
**Time of recording**	Intraoperative	98 *	**Recording location**	Intracochlear	92 ^†^
	Intra- and postoperative	2 *		Intra- and extracochlear	11 ^†^
	Postoperative	15		Extracochlear	12
	* Intraoperative			^†^ *Intracochlear*	
	During insertion	70		CI apical electrode	48
	During and after insertion	6		Various CI electrodes	39
	After insertion	24		Custom electrode	14
				CI apical and various electrodes	2
***x*** **-axis used**	Insertion time	34	***y*** **-axis used**	Waveform amplitude	23
	Time waveform	19		Response magnitude	62
	Recording electrode	23		Phase	7
	Different steps	6		Stimulus frequency	1
	Insertion depth	13		Sound pressure level	1
	Tonotopic position	3		Participants	1
	Stimulus frequency	2		Two or more *y*-axes	20
	Sound pressure level	1			
	Number of intracochlear electrodes	1			
	Angle of rotation	1			
	Two *x*-axes	12			
**ECochG component**	CM/DIF/FFT F0	71	**Extra**	Audiogram included	2
	Rarefaction/condensation	3		Notes during insertion	16
	ANN/SUM	2		Noise floor included	7
	CAP	4		Exemplary waveforms	8
	SP	2		Color bar (amplitude)	4
	Two or more components	17		Subplots	66
	Sum of spectral peaks of the harmonics	14		Multiple curves	75
	Not further specified	2		3D visualization	2

CI, cochlear implant; CM, cochlear microphonics; ANN, auditory nerve neurophonics; CAP, compound action potential; SP, summation potential; DIF, difference curve; SUM, sum curve; FFT F0, fast Fourier transform of the fundamental frequency.

**Table 2:** Participants’ demographics, side of implantation, etiology of hearing loss, preoperative pure tone average (PTA), preservation of hearing after surgery, length of the cochlear duct, and the electrode array.

Participant	Age at Implantation (Years)	Sex	Side	Etiology	Preoperative PTA (dB HL)	Hearing Preservation (%)	Length of Cochlear Duct (mm)	Electrode Array	Angular Insertion Depth (°)
P1	65	Male	Left	Progressive SNHL, unknown cause	83.75	57.1	36.2	HiFocus Mid-Scala (Advanced Bionics)	374°
P2	59	Male	Left	Ménière’s disease	63.75	37.1	36.9	CI 622 (Cochlear)	410°
P3	83	Male	Left	Progressive SNHL S/P chronic inflammation of the middle ear	97.5	11.3	37.0	CI 612 (Cochlear)	362°

SNHL, sensorineural hearing loss; S/P, status post; PTA, pure tone average for 0.25–2 kHz.

## Data Availability

The exemplary data and the scripts presented in this study are openly available on GitHub at https://github.com/OtoBM.

## Supplementary Materials

The following supporting information can be downloaded at: https://www.mdpi.com/article/10.3390/jcm13123470/s1. Table S1: Features of the included articles and their corresponding figures from the systematic literature search. References appear in the reference list of the main text. Table S2: PRISMA 2020 Checklist.
