# Supplementary material for: ZH-ECochG Bode Plot: A Novel Approach to Visualize Electrocochleographic Data in Cochlear Implant Users
Source: J Clin Med. 2024 Jun 14;13(12):3470. doi: 10.3390/jcm13123470 (PMC11205027; doi:10.3390/jcm13123470)
Supplement: Supplementary file 1 [file jcm-13-03470-s001.zip › Table S1.pdf]

Table S1. Features of the included articles and their corresponding figures from the systematic literature search.

Source	Year	Title	Study Population	Figure Nr.	Time of recording	Recording location	ECochG Component	Graph y-axis	Graph x-axis	Extra
Acharya et al. [58]	2016	Using the Implant Electrode Array to Conduct Real-time Intraoperative Hearing Monitoring During Pediatric Cochlear Implantation: Preliminary Experiences.	children	1	Intraoperative - during electrode insertion	IC - CI apical electrode	CM	Amplitude (mV) and insertion depth (mm)	Time (ms) waveform	• Multiple curves: waveforms at different insertion depths
Adel et al. [59]	2021	Band-Limited Chirp-Evoked Compound Action Potential in Guinea Pig: Comprehensive Neural Measure for Cochlear Implantation Monitoring.	guinea pig	2	Intraoperative - during electrode insertion	EC - RW	CM + CAP	Relative amplitude	Insertion depth (mm)	• 3D-visualization: different stimuli (z-axis)<br>• Multiple curves: different components (CM and CAP) from different stimuli
Adunka et al. [20]	2010	Intracochlear recordings of electrophysiological parameters indicating cochlear damage.	gerbils	3	Intraoperative - during electrode insertion	IC - custom electrode	CM + CAP	Amplitude difference between RW and IC (% of RW)	Distance from round window (µm)	• Multiple curves: different components (CM and CAP)
Andonie et al. [60]	2023	Real-Time Feature Extraction From Electrocochleography With Impedance Measurements During Cochlear Implantation Using Linear State-Space Models.	adult	4	Intraoperative - during electrode insertion	IC - CI apical electrode	CM/DIF	Amplitude (µV)	Insertion time (s)	• Notes: Start and full insertion<br>• Multiple curves: two analysis methods<br>• Exemplary waveforms

Table S1. Features of the included articles and their corresponding figures from the systematic literature search.

Source	Year	Title	Study Population	Figure Nr.	Time of recording	Recording location	ECochG Component	Graph y-axis	Graph x-axis	Extra
										• Impedances over insertion time
Arweiler-Harbeck et al. [61]	2021	Digital Live Imaging of Intraoperative Electrocochleography - First Description of Feasibility and Hearing Preservation During Cochlear Implantation.	adults	5	Intraoperative - during electrode insertion	IC - CI apical electrode	CM	F0 amplitude of the difference (μV)	Insertion time (s)	• Notes: Start and full insertion
Barnes et al. [62]	2021	Electrocochleography in cochlear implantation: Development and future directions.	simulation data	6	Intraoperative - during electrode insertion	IC - CI apical electrode	CM	Amplitude (μV)	Insertion time (s)	• Multiple curves: stimulation frequencies
Baumhoff et al. [63]	2023	Summating Potential as Marker of Intracochlear Position in Bipolar Electrocochleography.	guinea pig	7	Intraoperative - during electrode insertion	IC - pairs of neighboring CI electrodes	SP	SP amplitude (normalized)	Distance from base (%)	• Subplots: For each stimulus frequency
Bayri Ulukan et al. [64]	2023	Intracochlear electrocochleography findings in cochlear implant recipients with auditory neuropathy spectrum disorder.	children	8	Postoperative	IC - every second CI electrode	CM	F0 amplitude of the difference (μV)	Recording electrodes	• Subplots: different participants
Bester et al. [9]	2017	Characterizing electrocochleography in cochlear implant	adults	9	Intraoperative - after electrode insertion	IC - every second CI electrode	DIF	DIF magnitude (prop. of max)	Recording electrodes	• Subplots: different participants

Table S1. Features of the included articles and their corresponding figures from the systematic literature search.

Source	Year	Title	Study Population	Figure Nr.	Time of recording	Recording location	ECochG Component	Graph y-axis	Graph x-axis	Extra
		recipients with residual low-frequency hearing.		10	Intraoperative - after electrode insertion	IC - every second CI electrode	DIF	1. Magnitude (prop. Of max) 2. DIF latency (ms)	Recording electrodes	• Multiple curves: different participants (mean +-SD) • Subplots: different components (DIF, SUM) and magnitude-latency
Bester and Weder et al. [65]	2020	Cochlear microphonic latency predicts outer hair cell function in animal models and clinical populations.	adults	11	Intraoperative - after electrode insertion	IC - every second CI electrode	CM	CM latency (ms)	Recording electrodes	• Multiple curves: different participants
Bester et al. [66]	2022	Electrocochleography triggered intervention successfully preserves residual hearing during cochlear implantation: Results of a randomised clinical trial.	adults	12	Intraoperative - during electrode insertion	IC - CI apical electrode	CM	CM amplitude (µV)	Insertion time (s)	• Subplots: different participants
				13	Intraoperative - during electrode insertion	IC - CI apical electrode	CM	CM amplitude (µV)	Insertion time(s)	• Subplots: different participants • Notes: intervention, 16mm in insertion, full insertion
Bester and Dalbert et al. [34]	2023	Electrocochleographic Patterns Predicting Increased Impedances	adults	14	Intraoperative - after electrode insertion	IC - every second CI electrode	CM	Amplitude (µV) and recording electrodes	Time (ms) waveform	• Multiple curves: waveforms at different

Table S1. Features of the included articles and their corresponding figures from the systematic literature search.

Source	Year	Title	Study Population	Figure Nr.	Time of recording	Recording location	ECochG Component	Graph y-axis	Graph x-axis	Extra
		and Hearing Loss after Cochlear Implantation.								recording electrodes • Subplots: different participants
				15	Intraoperative - after electrode insertion + postoperative (3 months)	IC - every second CI electrode	CM	normalized CM amplitude (dB) and latency (ms)	Recording electrodes	• Noise floor • Subplots: Different time of recording and different participants • Multiple curves: amplitude and latency
				16	Intraoperative - after electrode insertion + postoperative (3 months)	IC - every second CI electrode	CM	normalized CM amplitude (dB)	Recording electrodes	• Multiple curves: different time of recording (mean + standard error) • Subplots: Different subgroups
Buechner et al. [67]	2022	Clinical experiences with intraoperative electrocochleography in cochlear implant recipients and its potential to reduce insertion trauma and	adults	17	Intraoperative - during electrode insertion	IC - CI apical electrode	CM	Amplitude in μV	Insertion time (s)	• Noise floor • Subplots: different participants
				18	Intraoperative - during electrode insertion	IC - CI apical electrode	CM	Amplitude (dB re 1 μV) + phase (rad)	Insertion time (s)	• Noise floor • Subplots: different participants

Table S1. Features of the included articles and their corresponding figures from the systematic literature search.

Source	Year	Title	Study Population	Figure Nr.	Time of recording	Recording location	ECochG Component	Graph y-axis	Graph x-axis	Extra
		improve postoperative hearing preservation.								• Multiple curves: amplitude and phase
Buhle et al. [68]	2023	Expanding Understanding of Electrocochleography in Cochlear Implantation: Auditory Neuropathy Spectrum Disorder With Normal Pure Tone Average.	adult	19	Intraoperative - after electrode insertion	IC - every CI electrode	CM	Amplitude (µV)	Recording electrodes	
Calloway et al. [69]	2014	Intracochlear electrocochleography during cochlear implantation.	adults + children	20	Intraoperative - before and during electrode insertion	EC - RW and IC - custom electrode	Sum of first and second harmonics (FFT)	Response magnitude (dB re 0.1 µV)	Insertion depth (mm)	• Multiple curves: different participants<br>• Subplot: different subgroups
Campbell et al. [70]	2010	Correlation of early auditory potentials and intracochlear electrode insertion properties: an animal model featuring near real-time monitoring.	gerbils	21	Intraoperative - during electrode insertion	IC - custom electrode	CM + CAP	Amplitude compared to reference (RW) and depth (µm)	Trial (insertion steps)	• Multiple curves: different components (CM + CAP)<br>• Subplot: different participants
Campbell et al. [72]	2015	Cochlear response telemetry: intracochlear electrocochleography via cochlear implant neural response	adults	22	Postoperative	IC - various CI electrode	CM + ANN	1. Relative CM magnitude 2. relative CM phase (degree) 3.	Recording electrodes (apical, medial, basal)	• Multiple curves: different participants<br>• Subplot: different components

Table S1. Features of the included articles and their corresponding figures from the systematic literature search.

Source	Year	Title	Study Population	Figure Nr.	Time of recording	Recording location	ECochG Component	Graph y-axis	Graph x-axis	Extra
		telemetry pilot study results.						relative ANN magnitude		(Magnitude CM + CM phase + magnitude ANN)
				23	Postoperative	IC - various CI electrode	CAP	Amplitude (µV) and recording electrodes	Time (ms) waveform	• Multiple curves: waveforms at different recording electrodes
Campbell et al. [35]	2016	Intraoperative Real-time Cochlear Response Telemetry Predicts Hearing Preservation in Cochlear Implantation.	adults + children	24	Intraoperative - during electrode insertion	IC - CI apical electrode	CM	1. Insertion time (s) and amplitude 2. CM amplitude (µVrms)	1. Time (ms) waveform 2. Insertion time (s)	• Multiple curves: waveforms at different insertion time • Subplots: waveforms and Amplitude (FFT) for different participants
Campbell et al. [71]	2017	Electrophysiological evidence of the basilar-membrane travelling wave and frequency place coding of sound in cochlear implant recipients.	adults	25	Intraoperative – during electrode insertion	IC – CI apical electrode	CM/DIF	Amplitude and Insertion time (s)	Time (ms) waveform	• Multiple curves: waveforms at different insertion time • Subplot: different participants
				26	Intraoperative - after electrode insertion	IC - every second CI electrode	CM + SP	Amplitude and	Time (ms) waveform	• Multiple curves: waveforms at

Table S1. Features of the included articles and their corresponding figures from the systematic literature search.

Source	Year	Title	Study Population	Figure Nr.	Time of recording	Recording location	ECochG Component	Graph y-axis	Graph x-axis	Extra
								recording electrodes		different recording electrodes • Subplot: different components (CM + SP)
				27	Intraoperative - after electrode insertion	IC - every second CI electrode	CM	Relative CM onset delay (ms)	Electrode frequency place (kHz)	• Multiple curves: different participants and literature data
Choudhury et al. [21]	2011	Detection of intracochlear damage with cochlear implantation in a gerbil model of hearing loss.	gerbils	28	Intraoperative - during electrode insertion	IC - custom electrode	CM + CAP	Relative magnitude and depth (µm)	Trials and Insertion time (min)	• Multiple curves: different components (CM + CAP)
Dalbert et al. [73]	2015	Extra-and intracochlear electrocochleography in cochlear implant recipients.	adults	29	Intraoperative - after electrode insertion	IC - various CI electrode	Sum of first and second harmonics (FFT)	Normalized ECoG response	Recording electrodes	• Multiple curves: different participants • Subplots: different stimulus frequency • Noise floor
Dalbert et al. [10]	2018	Assessment of cochlear function during cochlear implantation by extra- and intracochlear electrocochleography.	adults	30	Intraoperative - during electrode insertion	EC - promontory	Sum of first and second harmonics (FFT)	Normalized ECoG response	Insertion steps	
				31	Intraoperative - during electrode insertion	IC - CI apical electrode	Sum of first and second	Normalized ECoG response)	Insertion depth	• Multiple curves: different participants

Table S1. Features of the included articles and their corresponding figures from the systematic literature search.

Source	Year	Title	Study Population	Figure Nr.	Time of recording	Recording location	ECochG Component	Graph y-axis	Graph x-axis	Extra
							harmonics (FFT)			
Dalbert et al. [11]	2019	Changes of Electrocochleographic Responses During Cochlear Implantation Presented at the Annual Meeting of ADANO 2016 in Berlin.	adults	32	Intraoperative - before during and after electrode insertion	EC - promontory	Sum of first and second harmonics (FFT)	Amplitude and insertion steps	Time (ms) waveform	• Multiple curves: waveform at different insertion steps<br>• Subplots: different participants
				33	Intraoperative - before during and after electrode insertion	EC - promontory	Sum of first and second harmonics (FFT)	Normalized ECoG response	Insertion steps	• Subplots: different participants
Dalbert et al. [37]	2020	Correlation Between Electrocochleographic Changes During Surgery and Hearing Outcome in Cochlear Implant Recipients: A Case Report and Systematic Review of the Literature.	adults	34	Intraoperative – before and during electrode insertion	EC - promontory	Sum of first and second harmonics (FFT)	1. Amplitude and insertion steps<br>2. Amplitude dB re 1μV	1. Time (ms) waveform<br>2. Insertion steps	• Multiple curves: waveform at different insertion steps
				35	Intraoperative – during and after electrode insertion	IC – various CI electrode	Sum of first and second harmonics (FFT)	1. Amplitude and insertion depth (mm)<br>2. Amplitude and recording electrodes	Time (ms) waveform	• Multiple curves: waveform at different insertion depth / recording electrodes
Dalbert et al. [74]	2021	Simultaneous Intra- and Extracochlear Electrocochleography	adults	36	Intraoperative - during electrode insertion	EC - promontory and IC -	DIF	Amplitude (μV) and	Time (ms) waveform	• Multiple curves: waveform at

Table S1. Features of the included articles and their corresponding figures from the systematic literature search.

Source	Year	Title	Study Population	Figure Nr.	Time of recording	Recording location	ECochG Component	Graph y-axis	Graph x-axis	Extra
		During Electrode Insertion.				custom electrode		insertion steps		different insertion depth<br>• Subplots: different participants
				37	Intraoperative - during electrode insertion	EC - promontory and IC - custom electrode	DIF + SUM	Normalized amplitude DIF/SUM	Insertion steps	• Multiple curves: different participants<br>• Subplots: recording location and component (DIF + SUM)
				38	Intraoperative - during electrode insertion	EC - promontory and IC - custom electrode	DIF	Phase (°)	Insertion steps	• Multiple curves: different participants<br>• Subplots: recording location
DeMason et al. [75]	2012	Electrophysiological properties of cochlear implantation in the gerbil using a flexible array.	gerbils	39	Intraoperative - during electrode insertion	EC - RW and IC - custom electrode	CM + CAP	Relative magnitude and depth (mm)	Trials and Insertion time (min)	• Multiple curves: different components (CM + CAP)<br>• Subplots: different stimulus frequency
Eichler et al. [76]	2024	Two different methods to digitally visualize continuous electrocochleography	adults	40	Intraoperative - during electrode insertion	IC - CI apical electrode	CM	Amplitude (µV)	Insertion time (s)	• Noise floor<br>• Subplots: different participants

Table S1. Features of the included articles and their corresponding figures from the systematic literature search.

Source	Year	Title	Study Population	Figure Nr.	Time of recording	Recording location	ECochG Component	Graph y-axis	Graph x-axis	Extra
		potentials during cochlear implantation: a first description of feasibility.								
Gawęcki et al. [77]	2022	Robot-Assisted Electrode Insertion in Cochlear Implantation Controlled by Intraoperative Electrocochleography—A Pilot Study.	adults	41	Intraoperative - during electrode insertion	IC - custom electrode	CM	ECochG response (μV)	Insertion time (s)	• Subplots: different participants
Giardina et al. [78]	2018	Response Changes during electrode insertion of a Cochlear Implant Using Extracochlear Electrocochleography.	adults + children	42	Intraoperative - during electrode insertion	EC - RW	Rarefaction or condensation	Amplitude (μV)	Time (ms) waveform and Insertion steps	• Subplots: different participants
				43	Intraoperative - during electrode insertion	EC - RW	Sum of stimulus frequency and higher harmonics (FFT)	Amplitude (dB re 1 μV)	Insertion distance (mm)	• Multiple curves: Different participants<br>• Subplots: different subgroups
				44	Intraoperative - during electrode insertion	EC - RW	Rarefaction or condensation	Amplitude (μV) and insertion steps	1. Time (ms) waveform<br>2. Phase in cycle	• Multiple curves: waveform at different electrodes inserted

Table S1. Features of the included articles and their corresponding figures from the systematic literature search.

Source	Year	Title	Study Population	Figure Nr.	Time of recording	Recording location	ECochG Component	Graph y-axis	Graph x-axis	Extra
Giardina et al. [12]	2019	Intracochlear Electrocochleography: Response Patterns During Cochlear Implantation and Hearing Preservation.	adults + children	45	Intraoperative - during electrode insertion	IC - CI apical electrode	Sum of fundamental frequency and next two harmonics (FFT)	1. Amplitude (μV) and insertion depth<br>2. Amplitude (dB re 1 μV)	1. Time (ms) waveform<br>2. Insertion depth (mm)	• Multiple curves: waveform at different insertion depth
				46	Intraoperative - during electrode insertion	IC - CI apical electrode	Sum of fundamental frequency and next two harmonics (FFT)	1. Change in Responses (dB)<br>2. Normalized change in response (μV / μV)	Insertion depth (mm)	• Multiple curves: Different participants<br>• Subplots: different subgroups
				47	Intraoperative - during electrode insertion	IC - CI apical electrode	Sum of fundamental frequency and next two harmonics (FFT)	Amplitude (dB re 1μV)	Insertion depth (mm)	• Exemplary waveforms + FFT<br>• Subplots: different participants
Greisiger et al. [79]	2024	Intraoperative Measured Electrocochleography and Fluoroscopy Video to Detect Cochlea Trauma.	adults	48	Intraoperative - during electrode insertion	IC - CI apical electrode	CM	1. Hearing level (dB)<br>2. CM amplitude (μV)	1. Frequency (Hz)<br>2. Insertion time (s)	• Subplots: different participants and corresponding pre and postoperative audiogram
Harris et al. [80]	2011	Preliminary results and technique for electrophysiological	adults + children	49	Intraoperative - during electrode insertion	EC - stapes	Not specified	Amplitude and different intensities	Time (ms) waveform and	• Multiple curves: waveform at

Table S1. Features of the included articles and their corresponding figures from the systematic literature search.

**Source**	**Year**	**Title**	**Study Population**	**Figure Nr.**	**Time of recording**	**Recording location**	**ECochG Component**	**Graph y-axis**	**Graph x-axis**	**Extra**
		intra-operative monitoring of residual hearing during cochlear implantation.							insertion steps	different intensities and insertion steps
Harris et al. [13]	2017	Patterns Seen During Electrode Insertion Using Intracochlear Electrocochleography Obtained Directly Through a Cochlear Implant.	adults + children	50	Intraoperative - during electrode insertion	IC - CI apical electrode	DIF	Amplitude (μV)	Record number (insertion time)	• Exemplary waveforms
Harris et al. [14]	2017	Real-Time Intracochlear Electrocochleography Obtained Directly Through a Cochlear Implant.	adults + children	51	Intraoperative - during electrode insertion	IC - CI apical electrode	CM	Amplitude and insertion time (s)	Time (ms) waveform	• Multiple curves: waveform at different insertion time
				52	Intraoperative - during electrode insertion	IC - CI apical electrode	CM	Amplitude (μV)	Insertion time (ms)	• Noise floor (error bar) • Subplots: different participants
				53	Intraoperative - during electrode insertion	IC – CI 4 electrodes	CM	Amplitude (μV)	Recording electrodes	• Subplots: different participants
Helmstaedter et al. [81]	2018	The Summating Potential Is a Reliable Marker of Electrode Position in Electrocochleography:	guinea pigs	54	Intraoperative - after electrode insertion	IC - custom electrode	CAP + SP + CM	Electrode contacts and amplitude (μV)	Time (ms) waveform and different stimuli frequencies	

Table S1. Features of the included articles and their corresponding figures from the systematic literature search.

Source	Year	Title	Study Population	Figure Nr.	Time of recording	Recording location	ECochG Component	Graph y-axis	Graph x-axis	Extra
		Cochlear Implant as a Theragnostic Probe.		55	Intraoperative - after electrode insertion	IC - custom electrode	SP + CM	Frequency (kHz) and recording electrodes	Sound pressure level (dB SPL)	• color bar: amplitude (μV) • Multiple curves: SP and CM on left and right side of plot
Henslee et al. [82]	2021	Development and Characterization of an Electrocochleography-Guided Robotics-Assisted Cochlear Implant Array Insertion System.	sheep	56	Intraoperative - during electrode insertion	IC - custom electrode	CM	ECochG amplitude average (μV)	Insertion Time (s)	• Subplots: different participants
Imsiecke et al. [83]	2020	Psychoacoustic and electrophysiological electric-acoustic interaction effects in cochlear implant users with ipsilateral residual hearing.	adults	57	Postoperative	IC - various CI electrode	Rarefaction and condensation	Amplitude and recording electrodes	Time (ms) waveform	• Multiple curves: waveform at different recording electrodes
Jwair et al. [84]	2023	Acute effects of cochleostomy and electrode-array insertion on compound action potentials in normal-hearing guinea pigs.	guinea pigs	58	Intraoperative - before and during electrode insertion	EC - RW niche	CAP	Amplitude (μV) and insertion steps	Time (ms) waveform	• Multiple curves: waveform at different insertion steps • Subplots: different participants and different stimulus frequency

Table S1. Features of the included articles and their corresponding figures from the systematic literature search.

Source	Year	Title	Study Population	Figure Nr.	Time of recording	Recording location	ECochG Component	Graph y-axis	Graph x-axis	Extra
				59	Intraoperative - before and during electrode insertion	EC - RW niche	CM	Amplitude (μV) and insertion steps	Time (ms) waveform	• Multiple curves: waveform at different insertion steps • Subplots: different participants and different stimulus frequency
Kashani et al. [85]	2024	Combining Intraoperative Electrocochleography with Robotics-Assisted Electrode Array Insertion.	adults	60	Intraoperative - during electrode insertion	IC - CI apical electrode	CM	FFT magnitude (μV)	Insertion time (s)	• Multiple curves: stimulation frequencies • Notes: different electrodes intracochlear
Koka et al. [24]	2018	Intra-Cochlear Electrocochleography During Cochear Implant Electrode Insertion Is Predictive of Final Scalar Location.	adults	61	Intraoperative - during electrode insertion	IC - CI apical electrode	CM	CM amplitude and CM Phase (radius)	Insertion time (sec)	• Notes: different electrodes intracochlear • Multiple curves: amplitude and phase
Lenarz et al. [86]	2020	Hearing Preservation With a New Atraumatic Lateral Wall Electrode.	adults	62	Intraoperative - during electrode insertion	IC - CI apical electrode	CM	Amplitude (μV)	Insertion time (s)	
Lenarz et al. [39]	2022	Relationship Between Intraoperative Electrocochleography	adults	63	Intraoperative - during electrode insertion	IC - CI apical electrode	DIF	ECochG amplitude (μV)	Insertion time (s)	• Subplots: different participants

Table S1. Features of the included articles and their corresponding figures from the systematic literature search.

Source	Year	Title	Study Population	Figure Nr.	Time of recording	Recording location	ECochG Component	Graph y-axis	Graph x-axis	Extra
		and Hearing Preservation.								
Linder et al. [87]	2022	A Comparison of ECochG With the Subjective Sound Perception During Cochlear Implantation Under Local Anesthesia-A Case Series Study.	adults	64	Intraoperative - during electrode insertion	IC - CI apical electrode	DIF	Amplitude (μV)	Insertion time (s)	• Notes: different electrodes inserted + events
				65	Intraoperative - during electrode insertion	IC - CI apical electrode	DIF	ECochG response amplitude (μV)	Number of intracochlear electrodes	• Multiple curves: different participants
Lo et al. [88]	2018	Intraoperative force and electrocochleography measurements in an animal model of cochlear implantation.	guinea pigs	66	Intraoperative - during electrode insertion	IC - custom electrode	CM + CAP	ECochG magnitude (μV) and Force (mN)	Insertion time (s)	• Multiple curves: stimulus frequency and different components (CM, CAP, Force)
Lorens et al. [89]	2019	Cochlear Microphonics in Hearing Preservation Cochlear Implantees.	adults	67	Postoperative	IC - various CI electrode	CM	Amplitude (μV) and recording electrodes	Time (ms) waveform	• Multiple curves: waveform at different recording electrodes<br>• Subplots: different participants
Mandalà et al. [90]	2012	Electrocochleography during cochlear implantation for hearing preservation.	adults	68	Intraoperative - before during and after electrode insertion	EC - close to RW	CAP	1. Normalized amplitude decrease (%)	Insertion steps	• Subplots: amplitude and phase

Table S1. Features of the included articles and their corresponding figures from the systematic literature search.

Source	Year	Title	Study Population	Figure Nr.	Time of recording	Recording location	ECochG Component	Graph y-axis	Graph x-axis	Extra
								2. Latency shift (ms)		
Min et al. [91]	2022	Forward Electric Stimulation-Induced Interference in Intracochlear Electrocochleography of Acoustic Stimulation in the Cochlea of Guinea Pigs.	guinea pigs	69	Intraoperative - after electrode insertion	IC - custom electrode	CAP	CAP amplitude (µV)	Recording electrodes	• Multiple curves: different participants
O'Connell et al. [40]	2017	Intra- and postoperative electrocochleography may be predictive of final electrode position and postoperative hearing preservation.	adults	70	Intraoperative - during electrode insertion	IC - CI apical electrode	CM	CM amplitude (dB)	Insertion time (s)	• Multiple curves: different participants<br>• Subplots: subgroup
O'Leary et al. [41]	2020	Intraoperative Observational Real-time Electrocochleography as a Predictor of Hearing Loss After Cochlear Implantation: 3 and 12 Month Outcomes.	adults	71	Intraoperative - during electrode insertion	IC - CI apical electrode	CM	CM amplitude (µV)	Insertion time (s)	• Subplots: different participants<br>• Notes: start and full insertion<br>• Exemplary waveforms
O'Leary et al. [15]	2023	Monitoring Cochlear Health With Intracochlear Electrocochleography During Cochlear	adults	72	Intraoperative - during electrode insertion	IC - CI apical electrode	CM	CM amplitude (µV)	Insertion time (s)	• Subplots: different participants<br>• Exemplary waveforms

Table S1. Features of the included articles and their corresponding figures from the systematic literature search.

Source	Year	Title	Study Population	Figure Nr.	Time of recording	Recording location	ECochG Component	Graph y-axis	Graph x-axis	Extra
		Implantation: Findings From an International Clinical Investigation.								
Panario et al. [92]	2023	Characteristics of the Summating Potential Measured Across a Cochlear Implant Array as an Indicator of Cochlear Function.	adults	73	Intraoperative - after electrode insertion	IC - every second CI electrode	CM + ANN + SP	Amplitude (μV) and recording electrodes	Time (ms) waveform	• Subplots: different components (CM, ANN, SP) and subgroups<br>• Multiple curves: waveform at different recording electrodes
				74	Intraoperative - after electrode insertion	IC - every second CI electrode	CM + ANN + CAP + SP	Magnitude (relative amplitude)	Recording electrodes	• Subplots: different components (CM, ANN, SP) and subgroups<br>• Multiple curves: different participants
				75	Intraoperative - after electrode insertion	IC - every second CI electrode	SP	SP deflection	Recording electrodes	• Subplots: different participants
Polak et al. [93]	2022	In Vivo Basilar Membrane Time Delays in Humans.	adults	76	Postoperative	IC - CI electrodes closest to characteristic frequency	CM	Travelling wave delay (ms)	Angle of rotation (°)	• Exemplary raw data

Table S1. Features of the included articles and their corresponding figures from the systematic literature search.

Source	Year	Title	Study Population	Figure Nr.	Time of recording	Recording location	ECochG Component	Graph y-axis	Graph x-axis	Extra
				77	Postoperative	IC - CI electrodes closest to characteristic frequency	CM	Time delay (ms)	Frequency (Hz)	• Subplots: different participants and literature data
				78	Postoperative	IC - CI electrodes closest to characteristic frequency	DIF	Amplitude (μV) and recording electrodes (freq)	Time (ms) waveform	• Multiple curves: waveform at different recording electrodes
Ramos-Macias et al. [94]	2019	Intraoperative Intracochlear Electrocochleography and Residual Hearing Preservation Outcomes When Using Two Types of Slim Electrode Arrays in Cochlear Implantation.	adults	79	Intraoperative - during electrode insertion	IC - CI apical electrode	CM	1. Amplitude 2. Insertion time (s)	1. Insertion time (s) 2. Time (ms) waveform	• Subplots: different participants
Saoji et al. [25]	2019	Multi-frequency Electrocochleography Measurements can be Used to Monitor and Optimize Electrode Placement During Cochlear Implant Surgery.	adults	80	Intraoperative - during electrode insertion	IC - CI apical electrode	CM	Amplitude (μV)	Insertion time (min)	• Multiple curves: stimulation frequencies • Notes: Advancing and retracting electrode
Saoji et al. [95]	2022	Relationship Between Intraoperative Electrocochleography	adults	81	Intraoperative - during electrode insertion	IC - CI apical electrode	CM	Amplitude (μV)	Samples (insertion time)	• Subplots: different participants

Table S1. Features of the included articles and their corresponding figures from the systematic literature search.

Source	Year	Title	Study Population	Figure Nr.	Time of recording	Recording location	ECochG Component	Graph y-axis	Graph x-axis	Extra
		Responses and Immediate Postoperative Bone Conduction Thresholds in Cochlear Implantation.								• Notes: full insertion
Saoji et al. [17]	2023	Multi-Frequency Electrocochleography and Electrode Scan to Identify Electrode Insertion Trauma during Cochlear Implantation.	adults	82	Intraoperative - during electrode insertion	IC - CI apical electrode	CM	Amplitude (μV)	Insertion time (min)	• Multiple curves: stimulation frequencies • Subplots: different participants • Notes: Advancing and retracting electrode
				83	Intraoperative - after electrode insertion	IC - every CI electrode	CM	Amplitude (μV)	Recording electrodes	• Multiple curves: stimulation frequencies • Subplots: different participants
				84	Intraoperative - after electrode insertion	IC - every CI electrode	CM	Phase (cycles)	Recording electrodes	• Multiple curves: stimulation frequencies
Scheperle et al. [96]	2023	Evaluation of Real-Time Intracochlear Electrocochleography	adults	85	Intraoperative - during electrode insertion	IC - initial CI apical	DIF	Amplitude (μV)	Insertion time (s)	• Multiple curves:

Table S1. Features of the included articles and their corresponding figures from the systematic literature search.

Source	Year	Title	Study Population	Figure Nr.	Time of recording	Recording location	ECochG Component	Graph y-axis	Graph x-axis	Extra
		for Guiding Cochlear Implant Electrode Array Position.				electrode, then switch				recording electrode<br>• Subplots: different participants
Schuerch et al. [97]	2022	Increasing the reliability of real-time electrocochleography during cochlear implantation: a standardized guideline.	adults	86	Intraoperative - after electrode insertion	IC – CI apical electrode	CM	Amplitude (μV) and intracochlear electrodes	Time (ms) waveform	• Multiple curves: Waveforms at different amount of intracochlear electrodes
Schuerch et al. [98]	2022	Performing Intracochlear Electrocochleography during Cochlear Implantation.	adults	87	Intraoperative - during and after electrode insertion	IC - CI apical electrode, four CI electrodes	Not specified	1. Amplitude (μV) and insertion depth<br>2. Amplitude (μV) and recording electrodes	Time (ms) waveform	• Multiple curves: Waveforms at different insertion depth and recording electrodes<br>• Subplots: insertion depth and recording electrodes
Schuerch et al. [99]	2023	Objective evaluation of intracochlear electrocochleography: repeatability, thresholds, and tonotopic patterns.	adults	88	Postoperative	IC – CI 4 electrodes	CM	Stimulus frequency (Hz)	Tonotopic position (Hz)	• Color bar: amplitude<br>• Multiple curves: different participants
				89	Postoperative	IC – CI 4 electrodes	CM	Amplitude	Tonotopic position (Hz)	• Multiple curves: different participants

Table S1. Features of the included articles and their corresponding figures from the systematic literature search.

Source	Year	Title	Study Population	Figure Nr.	Time of recording	Recording location	ECochG Component	Graph y-axis	Graph x-axis	Extra
										• Subplots: stimulus frequency
Sijgers et al. [18]	2021	Simultaneous Intra- and Extracochlear Electrocochleography During Cochlear Implantation to Enhance Response Interpretation.	adults	90	Intraoperative - during electrode insertion	EC - near RW and IC - CI apical electrode	DIF	Amplitude re 1uV (dB)	Insertion depth (mm)	• Multiple curves: recording location • Subplots: different participants
				91	Intraoperative - during electrode insertion	EC - near RW and IC - CI apical electrode	DIF	Phase (degree)	Insertion depth (mm)	• Multiple curves: recording location • Subplots: different participants
				92	Intraoperative - during electrode insertion	EC - near RW and IC - CI apical electrode	DIF	Amplitude (μV) and Insertion depth (mm)	Time (ms) waveform	• Multiple curves: waveforms at different insertion depths • Subplots: different participants and recording location
				93	Intraoperative - during electrode insertion	EC - near RW and IC - CI apical electrode	SUM	Amplitude re 1uV (dB)	Insertion depth (mm)	• Multiple curves: different participants

Table S1. Features of the included articles and their corresponding figures from the systematic literature search.

Source	Year	Title	Study Population	Figure Nr.	Time of recording	Recording location	ECochG Component	Graph y-axis	Graph x-axis	Extra
										• Subplots: recording location
				94	Intraoperative - during electrode insertion	EC - near RW and IC - CI apical electrode	SUM	Amplitude (μV) and Insertion depth (mm)	Time (ms) waveform	• Multiple curves: waveforms at different insertion depths<br>• Subplots: different recording location
Sijgers et al. [42]	2023	Classification of Acoustic Hearing Preservation After Cochlear Implantation Using Electrocochleography.	adults	95	Intraoperative - during electrode insertion	EC - near RW and IC - CI apical electrode	DIF	1. Amplitude re 1uV (dB)<br>2. Phase (degree)	Insertion depth (mm)	• Multiple curves: recording location<br>• Subplots: amplitude-phase and different participants
				96	Intraoperative - during electrode insertion	IC - CI apical electrode	DIF + SUM	Amplitude re 1 μV (dB)	Insertion depth (mm)	• Multiple curves: different participants (mean +- SD)<br>• Subplots: different components (DIF, SUM) and subgroup

Table S1. Features of the included articles and their corresponding figures from the systematic literature search.

Source	Year	Title	Study Population	Figure Nr.	Time of recording	Recording location	ECochG Component	Graph y-axis	Graph x-axis	Extra
Skarżyński et al. [100]	2022	Multi-Frequency Intraoperative Monitoring of Hearing Preservation during Cochlear Implantation.	adult	97	Intraoperative - during electrode insertion	IC - CI apical electrode	Harmonics	1. Amplitude (μV)<br>2. Latency (ms)	Insertion time (s)	• Subplots: Amplitude and phase<br>• Exemplary waveforms<br>• Multiple curves: harmonics (stimuli = SPL Chirp)
Soulby et al. [101]	2021	Establishing Reproducibility and Correlation of Cochlear Microphonic Amplitude to Implant Electrode Position Using Intraoperative Electrocochleography and Postoperative Cone Beam Computed Tomography.	adults + children	98	Intraoperative - during electrode insertion	IC - CI apical electrode	CM	CM amplitude (μV)	Insertion time (s)	• Notes: Intracochlear electrode and full insertion<br>• Estimated position of the electrodes
				99	Intraoperative - after electrode insertion	IC - various CI electrodes	CM	CM amplitude (μV)	Recording electrodes	• Noise floor
				100	Intraoperative - after electrode insertion	IC - various CI electrodes	CM	Different participants	Recording electrodes	• Color coded: amplitude (trafic light)
Tejani et al. [102]	2019	Impact of stimulus frequency and recording electrode on electrocochleography in Hybrid cochlear implant users.	adults	101	Postoperative	IC - various CI electrodes	CM/DIF + ANN/SUM	1. FFT magnitude (μV)<br>2. Phase (pi rad)	Recording electrodes	• Multiple curves: different participants<br>• Subplots: amplitude-phase and different component (CM, ANN) and

Table S1. Features of the included articles and their corresponding figures from the systematic literature search.

Source	Year	Title	Study Population	Figure Nr.	Time of recording	Recording location	ECochG Component	Graph y-axis	Graph x-axis	Extra
										stimulus frequency
				102	Postoperative	IC - various CI electrodes	CM/DIF + ANN/SUM	FFT magnitude (μV)	Stimulus frequency (Hz)	• Multiple curves: different participants<br>• Subplots: different component (CM, ANN) and recording electrodes
				103	Postoperative	IC - various CI electrodes	CM/DIF + ANN/SUM	Normalized amplitude	Recording electrodes	• 3D-visualization: different stimuli frequency (z-axis)<br>• Color bar: Amplitude<br>• Subplots: different component (CM, ANN
van Gendt et al. [103]	2020	Simulating intracochlear electrocochleography with a combined model of acoustic hearing and electric current spread in the cochlea.	Simulating data and adults	104	Postoperative	IC - every CI electrodes	CM	Amplitude (μV)	Recording electrodes	• Subplots: different participants/ simulation and stimulus frequency<br>• Multiple curves: stimulus intensity

Table S1. Features of the included articles and their corresponding figures from the systematic literature search.

Source	Year	Title	Study Population	Figure Nr.	Time of recording	Recording location	ECochG Component	Graph y-axis	Graph x-axis	Extra
Varghese et al. [104]	2024	Identifying Slim Modiolar Electrode Tip Fold-Over With Intracochlear Electrocochleography.	adults and children	105	Intraoperative - during electrode insertion	IC - CI apical electrode	FFT first harmonic	FFT Amplitude (μV)	Insertion time (s)	• Multiple curves: stimulus frequency<br>• Notes: start and full insertion<br>• Subplots: different participants
				106	Intraoperative - after electrode insertion	IC - every second CI electrode	FFT first harmonic	Amplitude (μV)	Recording electrodes	• Subplots: different stimulus frequencies and participants
Walia et al. [106]	2021	Hearing Preservation After Cochlear Reimplantation Using Electrocochleography: A Case Report.	adult	107	Intraoperative - during electrode insertion	IC - CI apical electrode	FFT first harmonic	Amplitude (μV)	Insertion time (s)	• Notes: events
Walia et al. [105]	2022	Early Hearing Preservation Outcomes Following Cochlear Implantation With New Slim Lateral Wall Electrode Using Electrocochleography.	adults	108	Intraoperative - during electrode insertion	IC - CI apical electrode	ECochG-TR	Normalized ECochG-TR	% of complete insertion	• Subplots: different participants<br>• Audiogram: Postoperative PTA shift on each subplot
Walia et al. [48]	2022	Electrocochleography and cognition are important predictors of speech perception outcomes in noise for	adults	109	Postoperative	IC - every second CI electrode	ECochG-TR	ECochG amplitude (μV)	Recording electrodes	• Multiple curves: stimulus frequency<br>• Exemplary waveforms and FFT

Table S1. Features of the included articles and their corresponding figures from the systematic literature search.

Source	Year	Title	Study Population	Figure Nr.	Time of recording	Recording location	ECochG Component	Graph y-axis	Graph x-axis	Extra
		cochlear implant recipients.								
Walia et al. [19]	2022	Is Characteristic Frequency Limiting Real-Time Electrocochleography During Cochlear Implantation?	adults	110	Intraoperative - during electrode insertion and after electrode insertion	IC - CI apical electrode	DIF	ECochG Amplitude (μV)	Insertion time (sec)	• Multiple curves: stimulus frequency<br>• Subplots: different participants<br>• Notes: start and full insertion
				111	Intraoperative - during electrode insertion	IC - CI apical electrode<br>IC - every second CI electrode	DIF	Amplitude (μV)	1. Insertion time (s)<br>2. Recording electrodes	• Multiple curves: stimulus frequency<br>• Subplots: different participants<br>• Cochlear diameter visualized on subplot<br>• Notes: start and full insertion
Walia et al. [107]	2023	Improved Cochlear Implant Performance Estimation Using Tonotopic-Based Electrocochleography.	adult	112	Intraoperative - after electrode insertion	IC - every second CI electrode	CM	Amplitude (μV) and recording electrodes	Time (ms) waveform and stimulus frequency	• Multiple curves: waveforms at different recording electrodes and stimuli frequencies

Table S1. Features of the included articles and their corresponding figures from the systematic literature search.

Source	Year	Title	Study Population	Figure Nr.	Time of recording	Recording location	ECochG Component	Graph y-axis	Graph x-axis	Extra
				113	Intraoperative - after electrode insertion	IC - every second CI electrode	CM	Amplitude of CM (μV)	Recording electrodes	• Multiple curves: stimulus frequency
Weder et al. [108]	2020	Toward a Better Understanding of Electrocochleography: Analysis of Real-Time Recordings.	adults	114	Intraoperative - during electrode insertion	IC - CI apical electrode	DIF	DIF μμV)	Insertion time (s)	• Notes: white marker on CI electrode
Wijewickrema et al. [109]	2022	Automatic analysis of cochlear response using electrocochleography signals during cochlear implant surgery.	adults	115	Intraoperative - during electrode insertion	IC - CI apical electrode	CM	dB re : 1 μV	Insertion time (s)	• Subplots: different participants

IC = intracochlear recording; EC = extracochlear recording; CI = cochlear implant; CM = cochlear microphonics; ANN = auditory nerve neurophonics; CAP = compound action potential; SP = summation potential; SD = standard deviation; DIF = difference curve; SUM = sum curve
